# Supplementary material for: Intermittent Use of Anti-Hormonal Agents for the Endocrine Therapy of Sex-Hormone-Dependent Breast and Prostate Cancer: A Protocol for a Systematic Review
Source: Int J Environ Res Public Health. 2022 Nov 22;19(23):15486. doi: 10.3390/ijerph192315486 (PMC9735951; doi:10.3390/ijerph192315486)
Supplement: Supplementary file 1 [file ijerph-19-15486-s001.zip › S2_Table.pdf]

Review Matrix on the Intermittent Use of Antihormonal Agents for the Endocrine Therapy of Malignancies: a Systematic Review

Reference & Purpose						Study Design & Participants				
**RefID** only for internal use	**Source** Database DOI	**Author(s), Title**	**Publication: Type Year Journal**	**Origin: Research Sample**	**Aims / Purpose**	**Study Design, Setting, Institution** (if applicable)	**Duration including last Follow-Up**	**Recruitment**	**Health Condition** (Inclusion criteria)	**Participant Demographics** (e.g. age, gender)
	http://dx.doi.org/10.1016/j.eururo.2015.10.007	Schulman C, Cornel E, Matveev V, et al. Intermittent versus continuous androgen deprivation therapy in patients with relapsing or locally advanced prostate cancer: A phase 3b randomised study (ICELAND)	Journal Article **2016** *Eur Urol*	Research: Belgium, The Netherlands, Russia, Finland, Czech Republic, France, Germany, UK, Poland, Spain, Belgium Sample: 20 European countries	Assess efficacy, side effects, QoL of intermittent AD versus continuous AD in patients with nonmetastatic relapsing or locally advanced PCa. relapsing or locally advanced PCa.	RCT No information given on setting and institutions. Multi-centre study (n=102)	6 months induction, randomized phase 42 months, intervention stopped 36 months after randomization follow-up at 6-months intervals for 18 months. Time between first patient in, last patient out 7.1 years.	Initiation 2006, last patient out 2013	Locally advanced PCa (T3-T4) or elevated or rising PSA levels (5 mg/ml) after RP or radiotherapy. Age≥18 and <80 yr, Gleason score 6, ECOG performance status score 0-2, and ≥5-year life expectancy.	No information on age Male
	http://dx.doi.org/10.1016/j.ejca.2006.08.020	Beex L, Rose C, Mouridsen H, et al. Continuous versus intermittent tamoxifen versus intermittent/alternated tamoxifen and medroxyprogesterone acetate as first line endocrine treatment in advanced breast cancer: An EORTC phase III study	Journal Article **2006** *Eur J Cancer*	Research: The Netherlands, Sweden, Denmark, Poland, Spain, Belgium. Sample: The Netherlands, Belgium, Austria, South Africa. Not clearly described, information is missing	To assess whether intermittent endocrine therapy with tamoxifen or alternated with medroxyprogesterone acetate (MPA), could prolong progression-free survival, time to resistance to tamoxifen and overall survival, compared with continuous treatment.	RCT No information given on setting and institutions. Multi-centre study	8 year follow-up, observation period > 10 years. At the time of final evaluation 236 of 276 patients had died	Inclusion period 1987 - 1997	Postmenopausal women with hormone receptor positive or unknown, advanced breast cancer with progressive evaluable disease. No prior endocrine therapy the previous 12 months.	Median age 66.3 years, range 38.5-89.7 Female

Note: AD androgen deprivation; ECOG Eastern Cooperative Oncology Group; OS overall survival; PCa prostate cancer; PFS progression free survival; PSA prostate specific antigen; RCT randomized controlled trial; RP radical prostatectomy; QoL quality of life,

Intervention & Control							Overall Assessment		
**Intervention Description & Time Course** (duration of intervals)	**Comparator or Control**	**Number Included at Baseline**	**Lost to Follow-Up & Reasons**	**Outcome(s)** (QoL instrument)	**Results**	**Side Effects**	**Author's Conclusion**	**Reviewer's Comments**	**Risk of Bias Tool & Estimate**
Induction 6 months: Leuprorelin acetate 22.5 mg 3-mo depot, bicalutamide 50 mg 1x daily for 1 month. Randomization: Intermittent AD mo 6-42, If serum PSA rose to 2.5 ng/ml, treatment restarted every 3 month (plus bicalutamide 50 mg for 1 mo) until PSA declined to 1 ng/ml	Continuos treatment from induction	701 randomized, 602 entering follow-up, 101 completing follow-up	131 withdrawals after randomization. 21 adverse events, 23 deaths, 27 withdrawal of consent, 10 loss to follow-up, 4 protocol violations, 45 others (PSA increase, disease progression, metastasis, change of therapy, RP)	Time to PSA progression, PSA progression-free survival (PFS), overall survival (OS), testosterone levels, performance status, and QoL (EORTC QLQ-C30 and PCa-specific module QLQ-PR25)	Median number of injections administered after randomisation: continuous AD 12 (range: 1-12), intermittent AD 3 (range: 1–10). No statistically significant or clinically relevant differences between groups for time to PSA progression, PSA PFS, OS, mean PSA levels over time, or QoL.	73.9% had one AE, 25,8% one or more serious Aes. Most common Aes: hot flushes, hypertension, constipation. No statistically significant difference between groups.	Intermittent and continuous AD showed comparable efficacy, tolerability, and QoL in patients with nonmetastatic locally advanced or relapsing PCa. Potential benefits include reduced drug acquisition costs with comparable OS rates in intermittent AD.	Missing information on setting and institutions, age of participants	
Tamoxifen 40 mg daily for 4 mo prior to randomization with objective remission or stable disease. Arm 2 Tamoxifen alternating with no medication 2 months each; arm 3 Tamoxifen, no medication, MPA, no medication alternating for periods of two months each	Arm 1 continuous Tamoxifen	347 randomized, 276 after 4 months	9 early death, 10 early progression before 4 months, 16 refused randomization, 10 protocol violation, 2 lost to follow-up, 15 unknown	Time to resistance (TTR) to Tamoxifen, progression free survival (PFS), overall survival OS)	Resistance established in 84%, 70%, 55% in arms 1, 2, 3. Median TTR was 12.5 (9.1–21.1), 13.2 (8.8–19.8) 24.0 (16.9–61.0) months ($p < 0.001$) Median PFS was 11.0 (8.1–15.2), 8.0 (6.2–12.4) 10.8 (7.1–16.7) months (non-significant) Median OS was 35.1 (28.2–44.8), 35.2 (23.7–41.3) and 31.4 (25.6–53.9) months, (non-significant)	No severe side effects recorded. Percentage of side effects 5.4, 4.9, and 13.7 in arms 1, 2, 3, ($p < 0.001$) Flare-up phenomena were recorded in 3.5%, 2.8% and 3.5%.	Efficacy of intermittent and intermittent/alternating strategies is neither superior nor inferior to continuous tamoxifen therapy. Intermittent and intermittent/alternating endocrine treatment strategies in advanced breast cancer can safely be applied.	Missing information on origin of the sample, setting and institutions, number of centres	

Note: AD androgen deprivation; ECOG Eastern Cooperative Oncology Group; OS overall survival; PCa prostate cancer; PFS progression free survival; PSA prostate specific antigen; RCT randomized controlled trial; RP radical prostatectomy; QoL quality of life,
